# Selective pressurized liquid extraction of plant secondary metabolites: *Convallaria majalis* L. as a case

**DOI:** 10.1016/j.acax.2020.100040

**Published:** 2020-02-24

**Authors:** Xiaomeng Liang, Nikoline Juul Nielsen, Jan H. Christensen

**Affiliations:** Department of Plant and Environmental Sciences, University of Copenhagen, Thorvaldsensvej 40, 1871, Frederiksberg C, Denmark

**Keywords:** Selective pressurized liquid extraction, Plant secondary metabolites, Matrix effects, Natural toxins

## Abstract

A fast and efficient selective pressurized liquid extraction (sPLE) method was developed to extract secondary metabolites from complex plant matrix. *Convallaria majalis* L., a plant producing toxic steroids, was used as proof-of-concept. The method was optimized in the aspects of preheating, dispersant, extraction temperature and solvent, and the use of C18 as in-cell cleanup sorbent. Eight authentic natural steroids with diverse sugar moieties and hydrophobicities were selected as reference analytes and spiked to 0.1 g dried leaves. The extraction performance was evaluated based on the analytes’ stability, recovery, matrix effect in the electrospray interface and the level of co-extractives. With the optimal method, the extraction was finished in 10 min. A colorless extract was obtained with recoveries ranging from 63% to 107% and absolute matrix effects ranging from 3% to 27%. The optimized method was validated by extracting 0.1 g, 0.2 g and 0.4 g spiked plant samples; method accuracy and precision were assessed by recoveries and relative standard deviations of the combined extraction-analysis workflow. The method was also tested on soil samples and indicated its suitability for measuring secondary metabolites in multiple environmental matrices. To our knowledge, this is the first time sPLE has been reported to extract plant secondary metabolites from a complex plant matrix, with satisfactory recoveries and low matrix effects. This is also the first time (s)PLE has been reported to extract plant secondary metabolites from soil. We envision the method be coupled with liquid chromatography-electrospray ionization-high resolution mass spectrometry in a standard metabolomics workflow to facilitate plant metabolomics studies.

## Highlights

• A selective pressurized liquid extraction (sPLE) method to extract secondary metabolites from plant is described.
• The method is fast, efficient, selective, and in accordance with principles of green chemistry.
• Preheating the dry extraction cell resulted in degradation of digoxin.
• Sand and diatomaceous earth induced matrix effects in the electrospray source.
• The method complies with metabolomics workflows and is applicable to investigating plant secondary metabolites in the environment.

## Graphical abstract

**Figure:**
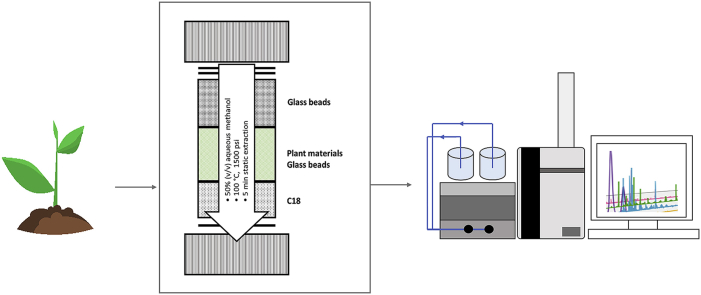

## 1 Introduction

Plant secondary metabolites are small natural compounds synthesized either as defensive chemicals against environmental stress, or as hormones mediating plant growth and development [1,2]. Since 1970s, a growing number of studies of plant secondary metabolites have been initiated; their chemical, biological and physiological properties, as well as medicinal values and environmental fate remain of interest to scientists till today [[3], [4], [5]]. Meanwhile, facilitated by developments in chromatography and mass spectrometry instrumentation (e.g. liquid chromatography-electrospray ionization-high resolution mass spectrometry (LC-ESI-HRMS)) and data mining strategies, more researchers have changed focus from analyzing a few compounds to a large profile, or even the global metabolome [6].

Sample preparation is the first step in plant metabolomics studies where the extraction process is a determining factor for data quality. Conventional extraction techniques such as Soxhlet extraction suffer from long extraction time and high solvent-consumption [7]. Alternative strategies such as ultrasound-assisted extraction (UAE) are advantageous in terms of simplicity but oftentimes have low recovery and require an additional cleanup step [8]. Ideally, an extraction method should be 1) simple and fast, 2) efficient and selective, 3) precise and 4) in accordance with the principles of green chemistry and EPA guidelines [9,10]. However, it is difficult to meet all these criteria in practice. This is mainly due to the high sample complexity, the presence of matrix interferences, low concentrations of the secondary metabolites and in some cases their unstable nature.

Pressurized liquid extraction (PLE) was first designed to extract persistent organic pollutants from soil but is also applicable to other solid matrices [[11], [12], [13], [14]]. Distinguished from the above methods, PLE operates at elevated pressure (500–3000 psi) and temperature (40–200 °C), leading to high recoveries due to enhanced mass transfer effects and disruption of the surface equilibria [11]. The method is also automated, time-efficient and environment friendly because of the relatively low solvent volumes. Moreover, since the extraction process is restricted in a sealed cell under inert atmosphere, it is well suited for metabolites, many of which are light sensitive and can be easily oxidized.

Attempts have been made to adapt PLE to the extraction of bioactive components from plant materials [[15], [16], [17], [18]]. However, only a few were compatible with LC-ESI-HRMS(MS). According to a recent review by Kharbach et al., among 189 published herbal extract screening studies in the year 2003–2019, only two used PLE as their sample preparation methods [19]. The reason for this could possibly be ascribed to the high co-extraction of chlorophylls and lipids [20,21]. The co-extractives may contaminate the chromatographic system, and may give rise to matrix effects in the electrospray interface, lower reproducibility and instrument long-term stability, and an increase in detection limits [22]. To date, a simple, efficient and selective extraction method compatible with LC-HRMS(MS) analysis and applicable to plant metabolomics studies is still lacking.

In this study, we aim to develop and validate a one-step selective PLE (sPLE) method which can be used to simultaneously extract secondary metabolites and remove potential interferences. A particular emphasis is given to the nonvolatile, (semi-)polar and toxic compounds which may leach from plant matrix to soil, migrate and contaminate water resources [23]. *Convallaria majalis* L., a popular urban invasive plant, served as proof-of-concept. The plant is distributed all over the northern hemisphere and was recently recognized as an emerging environmental pollution source for the toxic steroids produced [23,24]. To obtain as complete a metabolite profile as possible from the extraction, we selected eight authentic natural compounds from multiple steroid classes (cardenolide, bufadienolide, withanolide) with different sugar moieties (oligosaccharide, monosaccharide, aglycone) and hydrophobicities (log D 0.06 – 3.78 at pH 7.4, ChemAxon) (Table 1) as reference analytes throughout the study. The extraction performance is evaluated in terms of recovery and matrix effects for the following influencing factors: preheat time, extraction temperature, extraction solvent, dispersant and sorbent material. The optimized method is further validated in terms of method accuracy and precision, and will be tested for extraction of plant secondary metabolite in root soil.

**Table 1:** Chemical structures of the analytes in this study. All but BUF are authentic plant secondary metabolites. STR is the aglycone of CTX. DTG is the aglycone of ODR.

Compound (class)	CAS No.	Abbreviation	Formula	Log D (pH 7.4)^a^	Structure
Convallatoxin (cardenolide)	508-75-8	CTX	C_29_H_42_O_10_	0.06	
κ-Strophanthidin (cardenolide)	66-28-4	STR	C_23_H_32_O_6_	0.45	
Digoxin (cardenolide)	20830-75-5	DGX	C_41_H_64_O_14_	1.92	
Digitoxigenin (cardenolide)	143-62-4	DTG	C_23_H_34_O_4_	2.65	
Odoroside A (cardenolide)	12738-19-1	ODR	C_30_H_46_O_7_	3.46	
Proscillaridin (bufadienolide)	466-06-8	PRO	C_30_H_42_O_8_	2.30	
Bufalin (bufadienolide)	465-21-4	BUF	C_24_H_34_O_4_	3.28	
Withanolide A (withanolide)	32911-62-9	WTH	C_28_H_38_O_6_	3.78	

a Values predicted by ChemAxon.

## 2 Experimental

### 2.1 Samples

*Convallaria majalis* L. plants were collected from the Horticultural Garden of University of Copenhagen. Their identity was confirmed by Lars Birck, head gardener of the University. The leaves were briefly cleaned with distilled water, freeze-dried (Christ 1–4, Merck, Darmstadt, Germany) and milled (Cryomill, Retsch, Haan, Germany) into fine powder. Root soils were collected from where the plants were grown, air-dried and passed through a 2 mm sieve. All samples were stored in the dark at −20 °C until extraction.

### 2.2 Chemicals

κ-Strophanthidin (STR, > 90% purity), digitoxigenin (DTG, > 97% purity), odoroside A (ODA, > 97% purity), bufalin (BUF, > 98% purity), withanolide A (WTH, > 95% purity), proscillaridin (PRO, > 80% purity) and digoxin (DGX, > 95% purity) were purchased from Sigma-Aldrich (Hamburg, Germany). Convallatoxin (CTX) was purchased from MP Biomedicals (Irvine, CA, USA). Individual standard solutions were prepared in MeOH at concentration of 500 mg L^-1^ for STR, DGX, WTH and 100 mg L^-1^ for DTG, ODR, PRO, BUF, CTX and stored at −20 °C. A working standard mixture (ST) was prepared by 1000 times dilution in 50% (v/v) aqueous methanol prior to analysis.

LC-MS grade formic acid (FA), methanol (MeOH) and acetonitrile (ACN) were purchased from Sigma-Aldrich (Søborg, Denmark). Millipore water was obtained from a Millipore Milli Q-Plus system (Bedford, MA, USA). Glass beads (0.25 – 0.5 mm) were purchased from Carl Roth (Roth, Germany). C18 resin (Sepra E-C18, 50 μm) was purchased from Phenomenex (Værløse, Denmark). Diatomaceous earth and Ottawa sand (20 – 30 mesh) were purchased from Sigma-Aldrich (St. Louis, MI, USA) and Restek (Bellefonte, PA, USA); both were burnt overnight at 450 °C before use.

### 2.3 PLE

PLE was performed in an ASE 200 System (Dionex Corporation, Sunnyvale, USA) equipped with twelve 5 mL stainless steel extraction cells. Each cell was packed from bottom to top with: dispersant → sample-dispersant mixture → dispersant. For a sPLE, the dispersant at the bottom was replaced with 0.3 g C18 resin. Glass fiber filters (Advantec, Japan) were placed at both ends and between the layers. An aliquot of 100 μL of ST containing 0.5 mg L^-1^ STR, DGX, WTH and 0.1 mg L^-1^ DTG, ODR, PRO, BUF, CTX was spiked on top of the matrix unless otherwise specified. The cell was left for 0.5 h prior to extraction to allow a better incorporation of the spike solution into the matrix. The extraction was subjected to the following condition unless otherwise specified: preheat time: 0 min; static time: 5 min; cycles: 2; pressure: 1500 psi; flush volume: 70%; purge time: 60 s. Other parameters were varied based on the purpose of the experiment (Table 2). After an extraction, the crude extract was collected in an amber vial, final volume adjusted to 20 mL using the same extraction solvent. All extractions were performed in triplicates except those discussed section 3.1, which were performed in duplicates.

**Table 2:** An overview of variable PLE parameters and MS acquisition modes in this study.

	Sample weight (g)	In cell clean-up (sPLE)	Dispersant material	Extraction temperature (°C)	Extraction solvent (% MeOH, v/v)	MS mode^b^
Section 3.1	–	No	Ottawa sand	40, 60, 80, 100	50	SIR
Section 3.2	–	No	Ottawa sand, glass beads, diatomaceous earth	80	50	SIR
Section 3.3	–	No	Glass beads	40, 60, 80, 100	50	SIR
Section 3.4	0.1	Yes/No	Glass beads	60, 100	75, 50, 25, 0^a^	MRM
Section 3.5	0.1	Yes	Glass beads	60, 100	75, 50, 25, 0^a^	MRM
Section 3.6	0.1, 0.2, 0.4	Yes	Glass beads	100	50	MRM
Section 3.7	1.0 (soil)	Yes	Glass beads	100	50	MRM

a In the case of 0% MeOH, millipore water was used as extraction solvent.

b SIR – Selected Ion Recording (Selected Ion Monitoring); MRM –Multiple Reaction Monitoring.

To avoid carryover from re-use of an extraction cell, all parts of the cell were separated and thoroughly cleaned with water, sonicated successively in methanol and pentane-acetone mixture, and dried before a new sample was packed. In each extraction batch, a cell packed with non-spiked glass beads was included as method blank to confirm the system free of analyte contamination.

### 2.4 UV–vis

The absorbance spectra were acquired from a Lambda 25 double beam UV–*vis* spectrometer (PerkinElmer, USA). Scans were recorded from 350 to 750 nm at 25 °C.

### 2.5 LC-ESI-MS/MS

An aliquot of 10 μL of extract was injected on an UHPLC (Waters Acquity UPLC System, USA) equipped with an ACQUITY CSH C18 column (1.7 μm, 2.1 mm × 100 mm). The autosampler was set to 5 °C. Column temperature was set at 30 °C. The mobile phases were A: Milli-Q water +0.1% FA (v/v) and B: 95% ACN (v/v) with 0.1% FA (v/v). The flow rate was 0.4 mL min^-1^. The UHPLC program was: 1% B for 0.5 min, a linear gradient to 99% B in 5 min, isocratic elution for 2.5 min, ramping back to 1% B in 0.5 min, re-equilibration for 3.5 min. The total run time was 12 min.

MS acquisition was performed on a triple quadrupole (QqQ) mass spectrometer (Waters Micromass Quattro, USA) with electrospray ionization in positive mode. Selected Ion Recording (SIR) or Multiple Reaction Monitoring (MRM) was used (Table 2). This was because SIR was more sensitive than MRM when analysing samples composed of simple matrix (dispersing agent), whereas the opposite was the case when the sample contained complex matrix (plant and soil). The instrument parameters were set as follows: cone voltage: 30 V; cone gas: N_2_ (50 L h^-1^); source temperature: 100 °C; desolvation gas: N_2_ (400 °C, 800 L h^-1^); capillary voltage: 2.5 kV; scan time: 5 scan s^-1^; inter-scan delay time: 0.1 s. Detailed SIR and MRM acquisition parameters are listed in Table S1 (Supplemental information). MassLynx software version 4.1 was used for instrument control and data acquisition. Peak areas were integrated and used for quantification.

### 2.6 Recovery and matrix effect

To evaluate the extraction efficiency, a post-spiked solution (A_PS_) was prepared by spiking ST to a non-spiked extract and compared to a pre-spiked extract (A_ST_). The authentic non-spiked extract was regarded as background extract (A_BG_) and subtracted in the calculations (see Equation (1)):

(1) $$\text{Recovery }\left(\text{\%}\right)=\left(\frac{{\text{A}}_{\text{ST}}-{\text{A}}_{\text{BG}}}{{\text{A}}_{\text{PS}}-{\text{A}}_{\text{BG}}}\right)\;\times 100\text{\%}$$

To evaluate the matrix effects on the analytes, a new standard solution was prepared in the extraction solvent (A_SV_) and compared to the background (A_BG_) subtracted post-spike (A_PS_) at the same concentration level (see Equation (2)):

(2) $$\text{Matrix effect }\left(\text{\%}\right)=\left(\frac{{\text{A}}_{\text{PS}}-{\text{A}}_{\text{BG}}}{{\text{A}}_{\text{SV}}}\right)\;\times 100\text{\%}$$

## 3 Results and discussion

### 3.1 Preheating

**Fig. 1:**
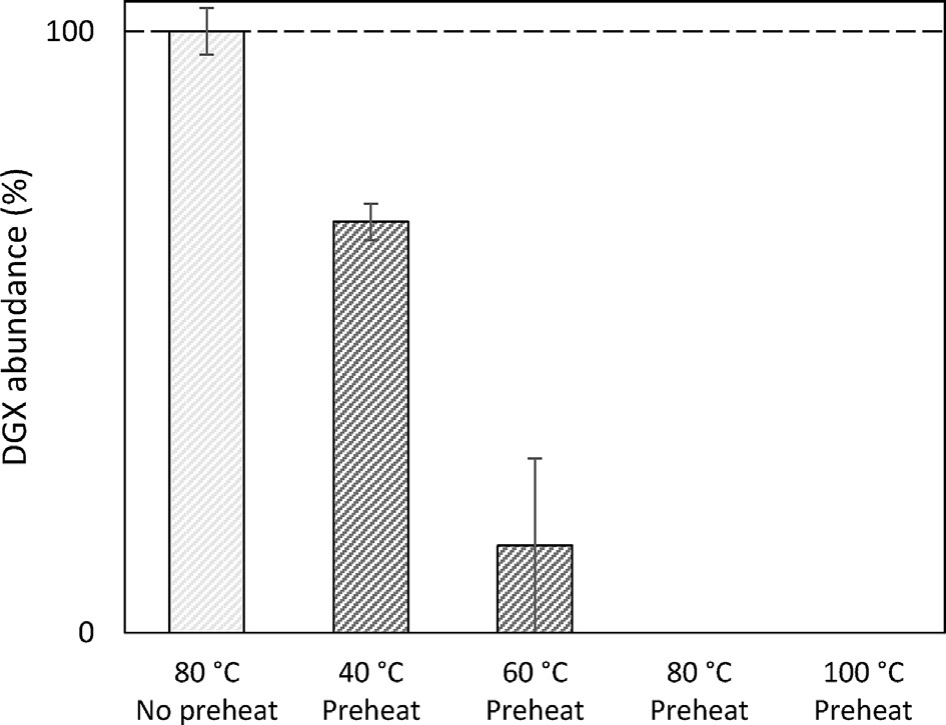
DGX recovered from spiked Ottawa sand. The extraction cells were either not preheated or preheated for 2 min. The error bars describe the standard deviation (n = 2).

As the first attempt, an aliquot of 100 μL ST-spiked Ottawa sand was extracted at 40, 60, 80 and 100 °C respectively with 50% MeOH. This was regarded as a pre-test of the thermal stabilities of the analytes during PLE. The cells were preheated in a dry state as recommended [25,26]. An unexpected and high loss of DGX was observed for all the extracts (Fig. 1). In a parallel extraction performed at 80 °C without preheating, however, there was no evidence of DGX loss. Therefore the degradation of DGX cannot solely be attributed to the elevated temperature. However, DGX can be adsorbed onto montmorillonite and hydrolyzed fast in solid state [27]. The observation of DGX degrading in a dry ASE cell indicates a similar behavior when this oligosaccharide interacts with other silicate-characterized substances, such as sand and diatomaceous earth, two of the most frequently used PLE dispersants. Consequently, the preheat step was eliminated in the subsequent investigations by opening the pump valve and introducing the extraction solvent into the extraction cell while the system was heating up [11].

### 3.2 Dispersing agent

**Fig. 2:**
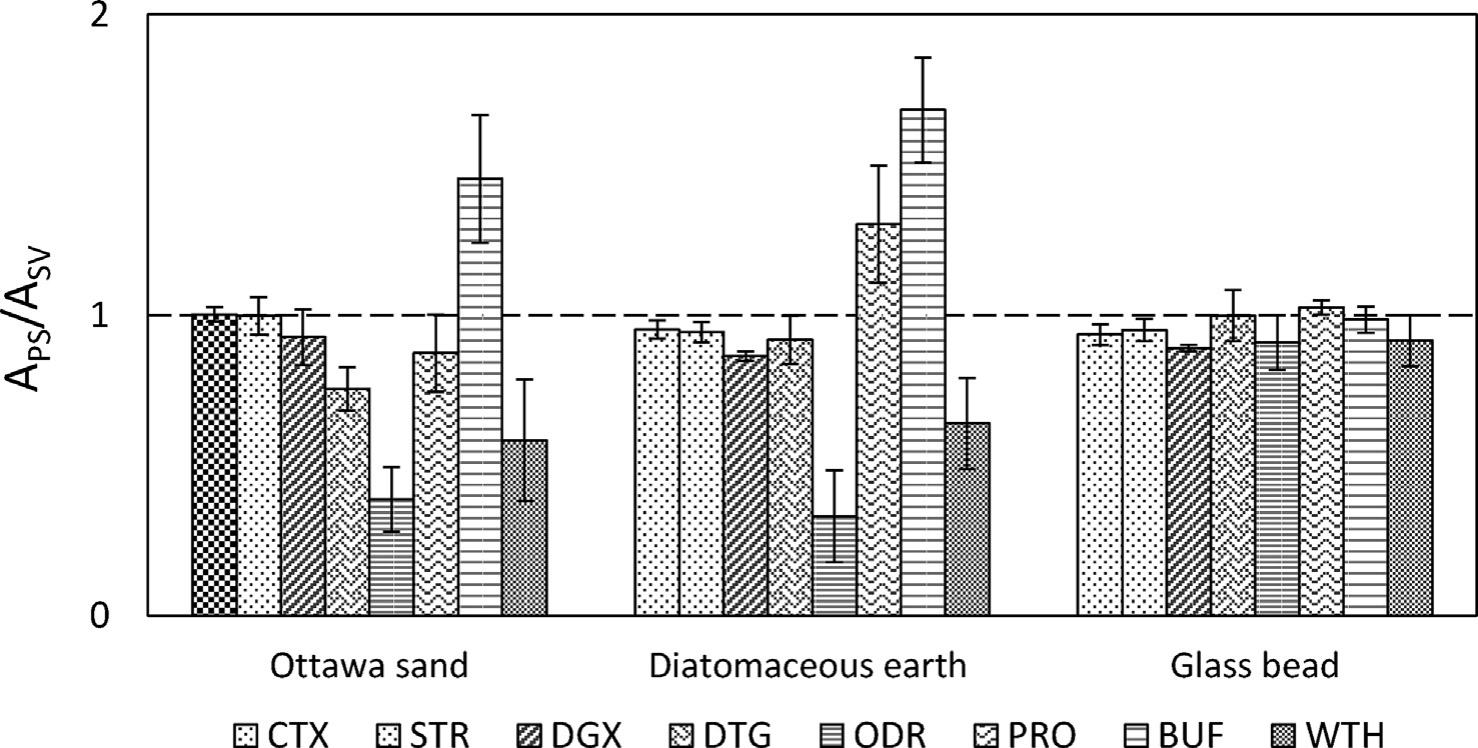
Matrix effects introduced by Ottawa sand, diatomaceous earth and glass beads. The error bars describe the standard deviation (n = 3).

In a standard ASE cell pack, the sample is oftentimes dispersed with inert materials to avoid aggregation and to facilitate the extraction process. Sand and diatomaceous earth are the two most frequently used dispersants for this purpose. Previously, the influence of different types of dispersants on recovering isoflavones from Spanish pulses was investigated by Delgado-Zamarreño et al.; hydromatrix (diatomaceous earth) afforded the best recoveries according to LC-UV analysis [28]. Here we included matrix effects in electrospray interface as an additional evaluation factor. For Ottawa sand, diatomaceous earth and glass beads, a 0.1 μg mL^-1^ post-spike solution was prepared in each background extract and compared to a standard solution at the same concentration level. Among the three, extracts of glass beads introduced the lowest matrix effects (Fig. 2). Extracts of sand and diatomaceous earth introduced matrix effects in a form of either signal suppression (DTG, ODR and WTH in both extracts, PRO in sand extract) or signal enhancement (BUF in both extracts, PRO in diatomaceous earth extract). Since all the three dispersants are composed of silica whereas sand and diatomaceous earth are acquired from nature, the matrix effects may result from some natural impurities which are thermally-resistant and extractable with the solvent. Accordingly, glass beads were chosen for method optimization.

### 3.3 Thermostability

**Fig. 3:**
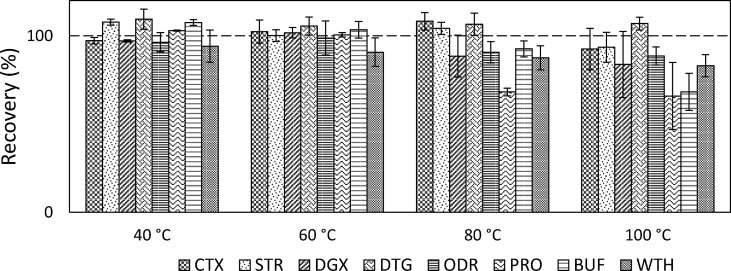
Thermostability of the secondary metabolites expressed by recovery when extracted at 40, 60, 80 and 100 °C. The error bars describe the standard deviation (n = 3).

An advantage of PLE for secondary metabolites is that, it operates in an inert atmosphere and therefore mitigates chemical degradation. To check the thermal stabilities of analytes during PLE, cells prefilled with glass beads were spiked with an aliquot of 100 μL ST and extracted at four temperatures ranging from 40 to 100 °C without preheating. A slight loss of the two bufadienolides, PRO and BUF, was observed at 80 and 100 °C respectively (Fig. 3). Nevertheless, this experiment was not based on a real sample matrix; the extraction efficiency which may increase along with the increased temperature therefore could not be evaluated. For this reason, the upper temperature limit (100 °C) remained in the subsequent investigation.

### 3.4 Sorbent

Crude plant extracts may contain abundant co-extractives, primarily being chlorophylls in the case of an extract from the leaves. A separate cleanup step has been required in order to eliminate these interferences and to achieve a better selectivity and instrument long-term stability (e.g. by percolating through a short column of activated charcoal or carbon). However, such manipulation is rather tedious and sometimes at the cost of losing analytes of interest [20].

**Fig. 4:**
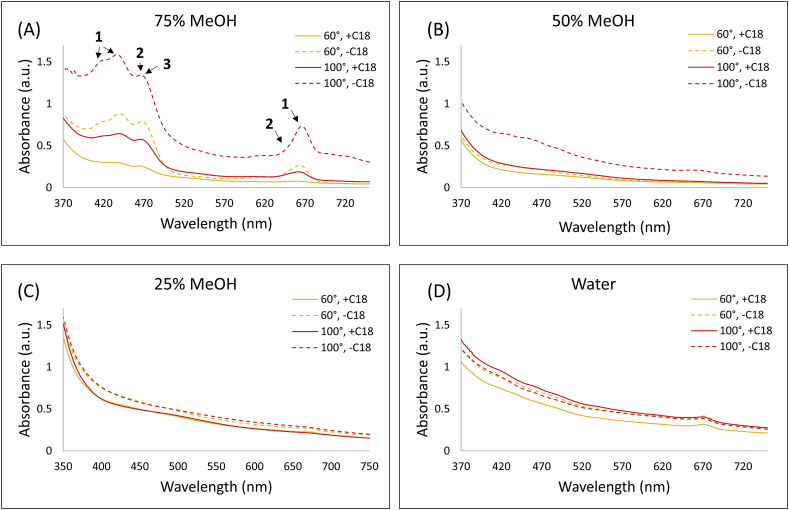
UV spectra of the plant extracts, either with C18 clean-up (+C18) or without (-C18). Extractions were performed in 75% (A), 50% (B), 25% MeOH (C) or in millipore water (D), at 60 °C (**–**) or 100 °C (**–**). Label **1** – chlorophyll A, **2** – chlorophyll B, **3** – β-carotene. Each plot was obtained from triplicate UV–*vis* records of the same sample.

In this study, the approach of sPLE was investigated by placing the sorbent at the bottom of a sample-packed extraction cell. Charcoal/carbon was replaced by C18, a material which was previously used to extract flavonoids from onions with satisfactory recoveries [29]. Apparently lighter plant extracts were obtained from those C18-included (+C18) cells (Fig. S1, supplementary information). The presence of three dominant pigments, chlorophyll A (**1**, λ_max_ at 415 nm, 432 nm and 665 nm) and chlorophyll B (**2**, λ_max_ at 469 nm and 652 nm) and β-carotene (**3**, λ_max_ at 470 nm) in the extracts obtained at temperatures of 60 °C and 100 °C and in different solvent mixtures (75, 50, 25% MeOH in water) was measured by UV–*vis* and confirmed by their characteristic maxima [30]. As shown in Fig. 4, extracts obtained in highly aqueous solvents (water and 25% MeOH) had a low chlorophyll or carotene content. While for 50% and 75% MeOH, the amount of co-extracted pigments increased especially for extracts where C18 were excluded (-C18), a significant drop of the absorbance was measured for those + C18 extracts, indicating an efficient removal of the chlorophylls and β-carotene by the C18 resin. For -C18 extracts using 50 and 75% MeOH at 100 °C (Fig. 4, A and B) the baseline increased, which could be an indication of scattering effects caused by particles that were soluble during the extraction but precipitated out while cooling down. These co-extractives were removed as well by C18 cleanup.

### 3.5 Method optimization and validation

**Fig. 5:**
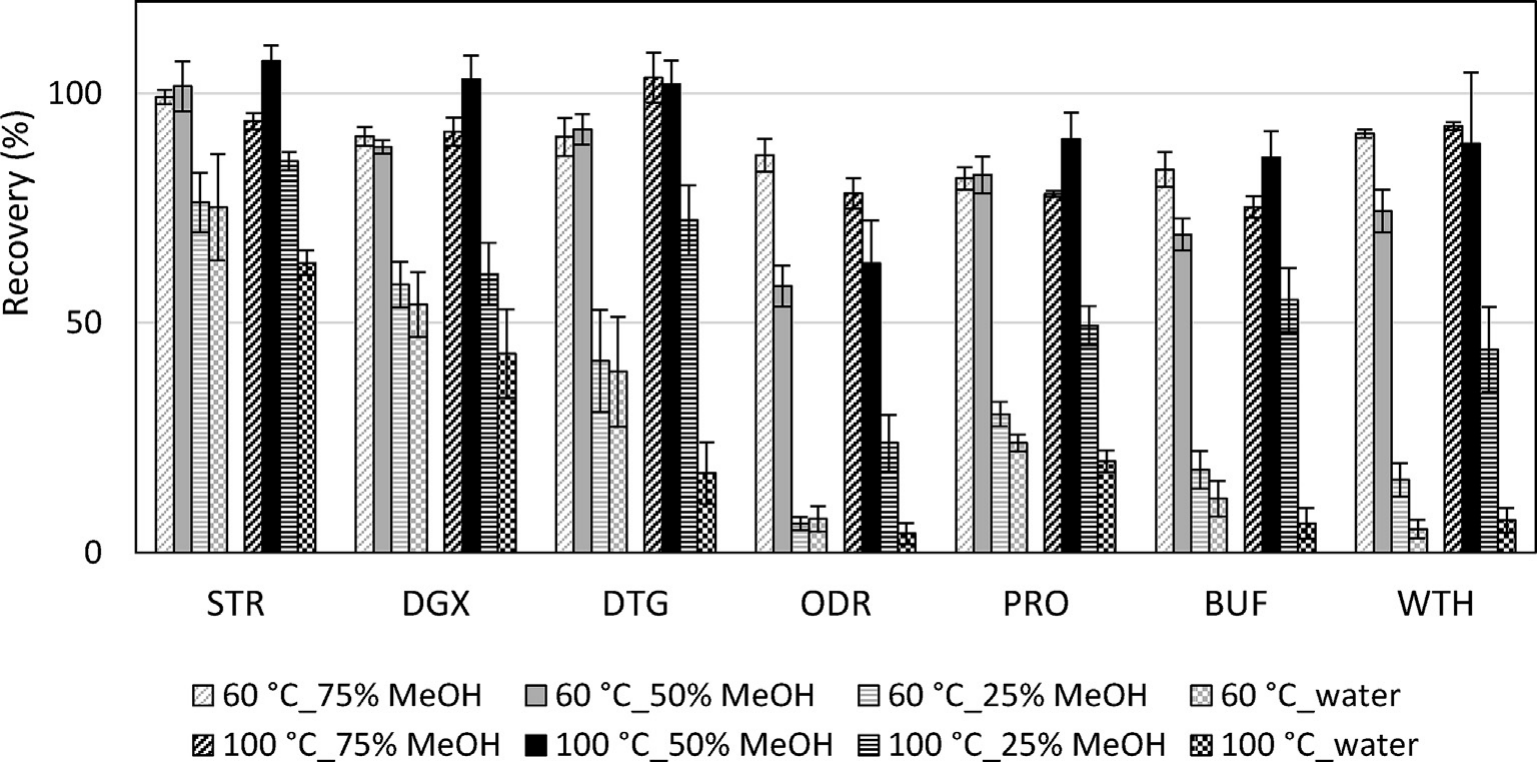
Recoveries of sPLE of secondary metabolites using 0, 25, 50 and 75% MeOH; 60 and 100 °C and C18 sorbent material. Matrix effects have been compensated in the calculations. The error bars describe the standard deviation (n = 3).

The sPLE conditions were further evaluated in terms of extraction recoveries and matrix effects by LC-MS analysis. CTX was not considered because of the insufficient spiked level compared to the high concentration in the sample extract. Thus the spiked quantity could not be reliably assessed [31]. As shown in Fig. 5, extraction in 50% MeOH at 100 °C provided the best recoveries in general (67–103%, Table 3). For PRO and BUF, which had showed their low stability at 100 °C (section 3.2), the loss caused by thermal degradation was compensated for by an increase in extraction efficiency at the elevated temperature.

**Table 3:** Recovery, precision (RSDs) and matrix effects of the secondary metabolites in plant and soil samples with the optimized sPLE method. Matrix effects have been compensated in recovery calculations.

Compound	Convallaria leaves							Root soil		
	0.1 g			0.2 g		0.4 g		1.0 g		
	Recovery (%); n = 3	RSD (%); n = 3	Matrix effect (%)	Recovery (%); n = 3	RSD (%); n = 3	Recovery (%); n = 3	RSD (%); n = 3	Recovery (%); n = 3	RSD (%); n = 3	Matrix effect (%)
CTX^a^	–	–	–	–	–	–	–	115	12	−5
STR	107	6	−3	116	5	117	7	76	18	29
DGX	103	9	3	114	8	112	9	119	3	−29
DTG	102	9	−27	108	7	110	11	96	3	26
ODR	63	16	−25	55	23	71	28	97	19	45
PRO	90	10	10	96	10	108	15	104	9	11
BUF	86	10	−5	90	13	96	15	126	2	40
WTH	89	27	−19	86	9	89	16	111	21	−56

a CTX in plant was not analyzed because of the very high endogenous levels which exceeded relevant spike levels.

**Fig. 6:**
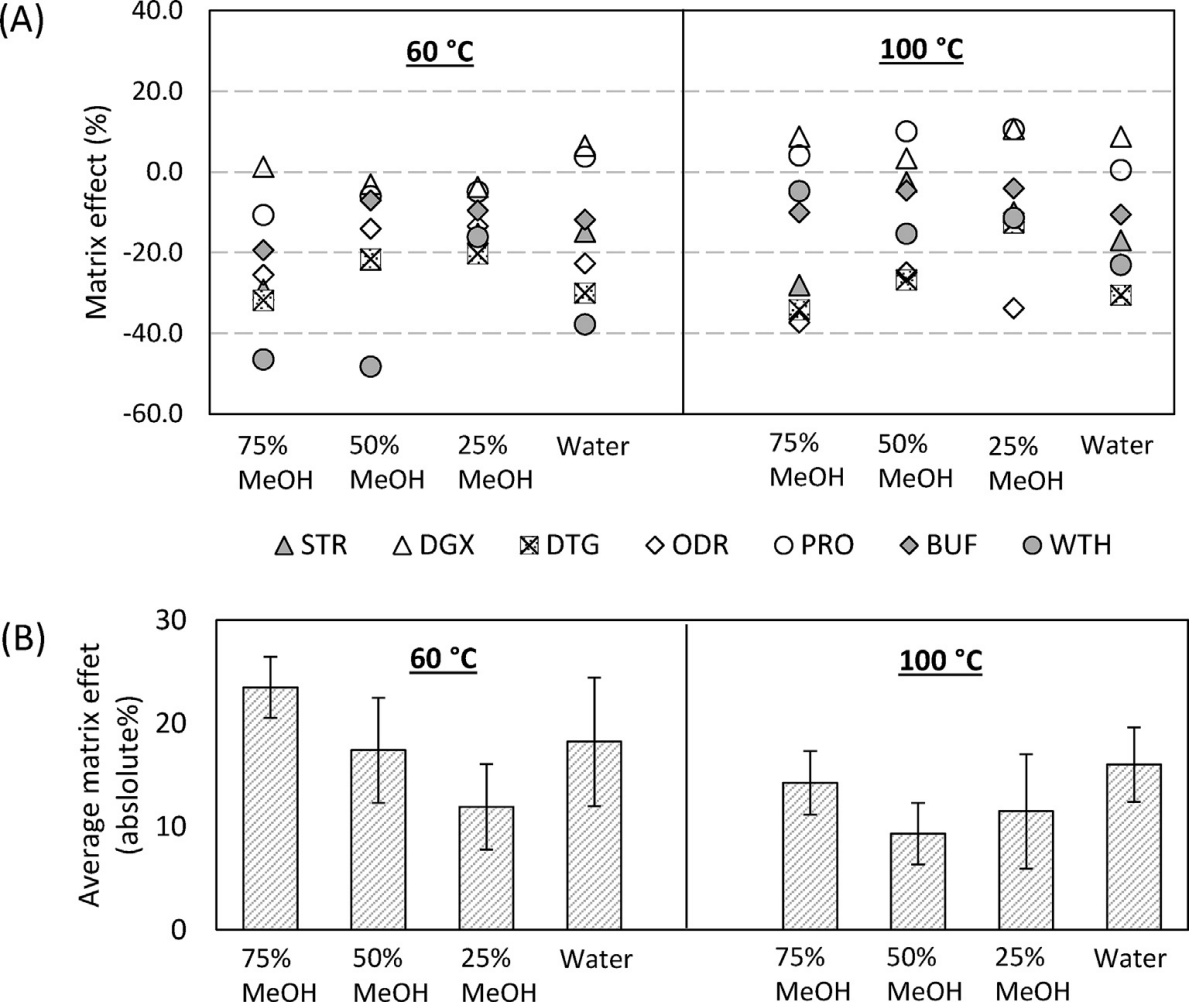
(A) Matrix effects induced by the co-extractives using 0, 25, 50 and 75% MeOH; 60 and 100 °C and C18 sorbent material. (B) Averaged matrix effects of each extraction condition, calculated based on absolute values. The error bars describe the standard deviation across 7 analytes and replicates (n = 3).

The matrix effects were evaluated individually for each reference compound. Signal suppressions were observed for all but DGX and PRO, both of which had enhanced signal responses in the water extract at 60 °C and all extracts at 100 °C (Fig. 6. A). The observation of opposite effects on the same analyte in the same extraction solvent but different temperatures (e.g. PRO, 50% MeOH, 60 °C and 100 °C) indicates the presence of different co-extractable substances in those extracts. As it turned out, the influence of temperature on sPLE selectivity was quite complicated as a result of the special extraction media (pressurized high diffusion liquids) and the sorbent (C18); the hypothesis that a higher temperature would yield more co-extractives thus higher matrix effects was not supported by the results. In general, the sPLEs at 100 °C had less interferences than at 60 °C (Fig. 6. B). The one performed in 50% MeOH at 100 °C exhibited the lowest matrix effects on average compared to the other conditions.

The preferred method was 50% MeOH and 100 °C because of the overall high recoveries and low matrix effects obtained. Parameters such as flush volume, pressure, static time and static cycle were not investigated, as they have previously been revealed to have limited effects on the extraction performance compared to the discussed variables [14,17,32]. The optimal extraction condition was therefore determined as: extraction temperature: 100 °C; extraction solvent: 50% MeOH; preheat time: 0 min; static time: 5 min; static cycle: 2; pressure: 1500 psi; flush volume: 70%; purge time: 60 s; in-cell cleanup using 0.3 g of C18 sorbent.

The optimized sPLE was validated by extracting 0.1, 0.2 and 0.4 g plant samples which were pre-spiked with an aliquot of 100 μL working solution prepared at concentration of 1 × ST, 2 × ST, 4 × ST respectively. Method accuracy was assessed by recoveries of which consistent values were obtained for each reference analyte: 107–117% for STR, 103–114% for DGX, 102–110% for DTG, 90–108% for PRO, 55–71% for ODR, 86–96% for BUF and 86–89% for WTH (Table 3). The precision was assessed by relative standard deviations (RSDs) of the sPLE method using LC-MS/MS for peak detection and quantification. RSDs were in a range from 5 to 16% with three exceptions: 27% for WTH (sample size 0.1 g), 23% and 28% for ODR (sample size 0.2 and 0.4 g). The high RSD values may result from two sources: work-up variation during the sPLE or analytical variation arising from the LC-MS instrument. In this particular case, the MS used was very old and therefore it was hypothesized that the majority of the variation originated from the MS operation, but not from the sPLE step. Quality control samples run before, within, and after the sequence confirmed that 10 out of the 16% RSD of ODR and 8 out of the 27% RSD for WTH originated from the MS variation (Table S2, supplementary information). Keeping this in mind, RSDs are acceptable. The limit of detection (LOD) and limit of quantification (LOQ) were estimated based on signal-to-noise ratio (S/N) of spiked sample (S/N_PS_ – S/N_BG_). The LOD (S/N = 3) ranged from 0.20 ng L^-1^ (BUF) to 15.8 ng L^-1^ (WTH), and the LOQ (S/N = 10) ranged from 0.66 ng L^-1^ (BUF) to 52.6 ng L^-1^ (WTH) (Table S1, supplementary information). These LODs and LOQs do not meet the state-of-the-art within the field, but should be sufficiently low for this sPLE study which is considered as the first step to establish an integrated plant metabolomics workflow.

### 3.6 Method comparison

In a study by Sonibare et al., Soxhlet extraction and sonication were employed to extract phenolic compounds from the leaves of *Musa acuminata* for metabolite profiling. For a 5 g dry leaf sample, both methods consumed 100 mL of 70% MeOH. The Soxhlet extraction took 9 h and gave 20–60% recoveries, while the sonication took 30 min and gave 18–23% recoveries [7]. As a comparison, our optimized sPLE method required smaller sample size (0.1 g), consumed less hazardous solvent (20 mL of 50% MeOH), and achieved a significant higher extraction efficiency in less time (10 min, 63–107% recoveries). The high extraction efficiency is in accordance with those reported in previous PLE studies [16,17,28]. Yet many of these studies emphasized on recoveries; the co-extracted interferences and the induced matrix effects in the electrospray interface were most often absent in the discussion sections. Romera-Torres et al. adapted a PLE method to selectively extract tropane alkaloids from feed; the PLE was followed by a solid phase extraction step and a chitosan cleanup, ending up with matrix effects of 16–41% [33]. Here our optimized sPLE method achieved a high level of selectivity as well as low matrix effects of 3–27%. Furthermore the manipulation was much simpler by combining the stepwise extraction and cleanup into one step. We recommend the incorporation of C18 to sPLE in plant and environment analysis to selectively extract polar secondary metaoblites (e.g. log D 0.06 – 3.78 covered in the study) but not pigments and organic matters, most of which are non-polar and expected to retain on the sorbent due to strong hydrophobic interaction.

### 3.7 Extended application (soil extraction)

Thereafter, the sPLE method was applied to extraction of root soil. 1.0 g of root soil sample was weighted, spiked and extracted the same way as the plant samples. As shown in Table 3, recoveries of the eight reference analytes were between 76% and 126%, absolute matrix effects were between 5% and 56%. Ion suppressions were observed for CTX, DGX and WTH; ion enhancements were observed for STR, DTG, ODR, PRO and BUG. The LOD ranged from 0.12 ng L^-1^ (DTG) to 25.0 ng L^-1^ (WTH), and the LOQ ranged from 0.40 ng L^-1^ (DTG) to 83.3 ng L^-1^ (WTH) (Table S1, supplementary information).

Currently, only a few documentations of plant secondary metabolites quantified in soil samples exist, therefore it is difficult to determine their environmentally relevant concentration [34]. However, considering the leaching, migration and degradation process, the concentration is expected to be at trace level and much lower than in any plant matrices. To this end, the high extraction yield obtained from this study is particularly important for a valid detection, identification and monitoring of secondary metabolites in the environment.

## 4 Conclusions

To our knowledge, this is the first time sPLE has been reported to extract plant secondary metabolites from a complex plant matrix, with satisfactory extraction recoveries (63–107%) and low matrix effects (3%–27%) achieved at the same time. RSDs of the integral sPLE-LC-MS/MS method was 5–16%. Detection limit was 0.20–15.8 ng L^-1^. The sPLE is compatible with LC-ESI-HRMS(MS) for large scale secondary metabolite profiling. This is also the first time (s)PLE has been reported to extract plant secondary metabolites from soil. We envision the method be coupled with LC-ESI-HRMS(MS) in a standard metabolomics workflow and facilitate future plant metabolomics studies.

## CRediT authorship contribution statement

**Xiaomeng Liang:** Conceptualization, Methodology, Software, Validation, Data curation, Formal analysis, Writing - original draft. **Nikoline Juul Nielsen:** Supervision, Writing - review & editing, Project administration. **Jan H. Christensen:** Supervision, Writing - review & editing, Funding acquisition.

## Declaration of competing interest

The authors declare that they have no known competing financial interests or personal relationships that could have appeared to influence the work reported in this paper.
