# Supplementary material for: Selective pressurized liquid extraction of plant secondary metabolites: Convallaria majalis L. as a case
Source: Anal Chim Acta X. 2020 Feb 24;4:100040. doi: 10.1016/j.acax.2020.100040 (PMC7587049; doi:10.1016/j.acax.2020.100040)

**Selective pressurized liquid extraction of plant secondary metabolites: *Convallaria majalis* L. as a case**

Xiaomeng Liang; Nikoline Juul Nielsen; Jan H. Christensen

*Department of Plant and Environmental Sciences, University of Copenhagen, Thorvaldsensvej 40, 1871, Frederiksberg C, Denmark*

**Table S1**

SIR and MRM acquisition parameters for each compound, as well as the limit of detection (signal-to-noise = 3) and LOQ (signal-to-noise = 10) measured at the MRM mode.

|  |  | **SIR** |  | **MRM** | | | **Leaves** | | **Soil** | |
| --- | --- | --- | --- | --- | --- | --- | --- | --- | --- | --- |
| **Analyte** | **t_R_ (min)** | **Selected ion (m/z)** |  | **Precusor ion (m/z)** | **Product ion (m/z)** | **Collision**  **(eV)** | **LOD* (ng L^-1^)** | **LOQ* (ng L^-1^)** | **LOD (ng L^-1^)** | **LOQ (ng L^-1^)** |
| CTX | 4.07 | 551 (M+H)^+^ |  | 551 (M+H)^+^ | 369 | 10 | 1.01 | 3.38 | 0.12 | 0.41 |
| STR | 4.26 | 405 (M+H)^+^ |  | 405 (M+H)^+^ | 369 | 10 | 6.91 | 23.0 | 2.95 | 9.84 |
| DGX | 4.46 | 803 (M+Na)^+^ |  | 781 (M+H)^+^ | 651 | 10 | 9.04 | 30.1 | 9.32 | 31.1 |
| DTG | 5.00 | 375 (M+H)^+^ |  | 375 (M+H)^+^ | 339 | 10 | 1.03 | 3.44 | 0.12 | 0.40 |
| ODR | 5.29 | 582 (M+ACN+Na)^+^ |  | 519 (M+H)^+^ | 375 | 10 | 0.36 | 1.20 | 0.28 | 0.93 |
| PRO | 4.79 | 531 (M+H)^+^ |  | 531 (M+H)^+^ | 531 | 10 | 1.81 | 6.02 | 2.16 | 7.19 |
| BUF | 5.23 | 387 (M+H)^+^ |  | 387 (M+H)^+^ | 387 | 10 | 0.20 | 0.66 | 0.28 | 0.94 |
| WTH | 5.25 | 493 (M+Na)^+^ |  | 471 (M+H)^+^ | 399 | 10 | 15.8 | 52.6 | 25.0 | 83.3 |

*LOD: limit of detection; LOQ: limit of quantification.

**Table S2**

Intraday precision of the two compounds, ODR and WTH measured on the LC-ESI-QqQ instrument using MRM mode. Both were prepared at 0.1 mg L^-1^ together with the other six compounds in a composite plant extract, regarded as the quality control check sample.

|  | **ODR** | | | **WTH** | | |
| --- | --- | --- | --- | --- | --- | --- |
| **Injection #** | **RT, min** | **Peak area** | **Peak height** | **RT, min** | **Peak area** | **Peak height** |
| 1 | 5.29 | 181 | 5174 | 5.25 | 153 | 4771 |
| 2 | 5.29 | 161 | 4544 | 5.25 | 151 | 4550 |
| 3 | 5.29 | 179 | 5821 | 5.26 | 160 | 4710 |
| 4 | 5.27 | 159 | 4774 | 5.25 | 149 | 4808 |
| 5 | 5.30 | 175 | 5246 | 5.25 | 170 | 4189 |
| 6 | 5.29 | 193 | 5380 | 5.25 | 187 | 5462 |
| 7 | 5.29 | 186 | 5535 | 5.25 | 172 | 4661 |
| 8 | 5.26 | 128 | 3324 | 5.25 | 163 | 4795 |
| 9 | 5.29 | 168 | 5216 | 5.27 | 151 | 4797 |
| 10 | 5.29 | 173 | 6072 | 5.25 | 135 | 3797 |
| 11 | 5.29 | 177 | 6466 | 5.25 | 162 | 4952 |
| 12 | 5.29 | 158 | 4101 | 5.25 | 153 | 4214 |
| 13 | 5.29 | 181 | 6046 | 5.25 | 150 | 3682 |
| 14 | 5.29 | 197 | 5938 | 5.25 | 138 | 3624 |
| 15 | 5.28 | 159 | 5427 | 5.25 | 164 | 5313 |
| **RSD (%)** | **0.2** | **10** | **16** | **0.1** | **8** | **12** |

**Fig S1.**

PLE with or without in-cell clean-up by C18 (selective PLE). For each pair, the left was obtained from sPLE, the right was from PLE without in-cell cleanup.


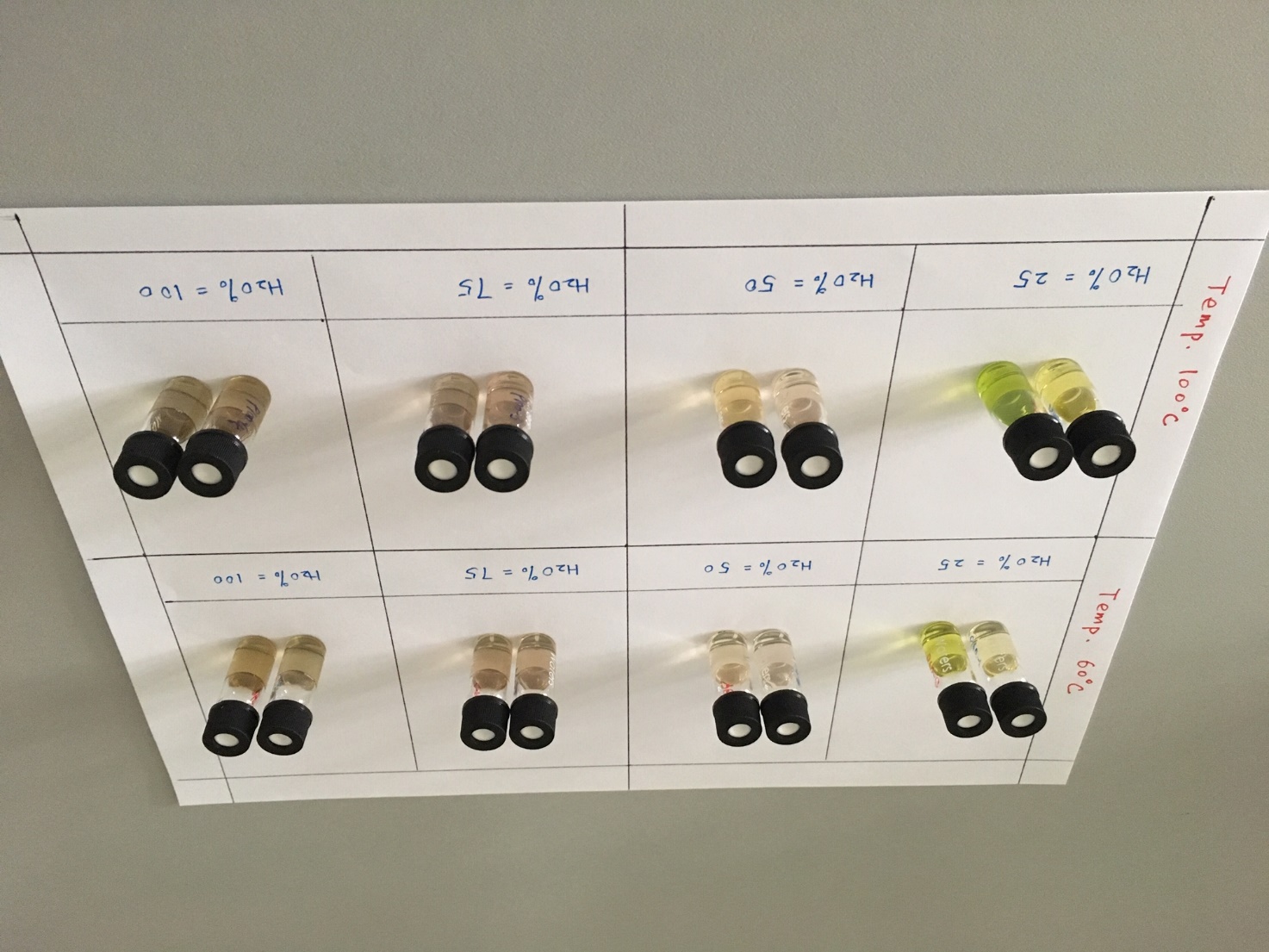

Supplement: Multimedia component 1 [file mmc1.docx]
